# Angiographic anatomy of the extracranial and intracranial portions of the internal carotid arteries in donkeys

**DOI:** 10.1186/s13620-017-0090-0

**Published:** 2017-04-20

**Authors:** Nurul Hayah Khairuddin, Martin Sullivan, Patrick J. Pollock

**Affiliations:** 1 Department of Farm and Exotic Animal Medicine and Surgery, Faculty of Veterinary Medicine, Universiti Putra Malaysia, 43400 UPM Serdang, Kuala Lumpur, Selangor Malaysia; 2 Small Animal Clinical Sciences, School of Veterinary Medicine, University of Glasgow, Glasgow, UK; 3 Weipers Centre Equine Hospital, School of Veterinary Medicine, University of Glasgow, Glasgow, UK

**Keywords:** Donkeys, Internal carotid artery, Rotational angiography

## Abstract

**Background:**

In horses, the extracranial and intracranial pathway of the internal carotid artery has been described. The extracranial pathway of the internal carotid artery begins at the carotid termination and runs on the dorsal surface of the medial compartment of the guttural pouch. Thereafter the internal carotid artery passes through the foramen lacerum to continue intracranially, forming part of the rostrolateral quadrants of the cerebral arterial circle (Circle of Willis). The objectives of this study were to define and record the anatomy of the carotid arterial tree and the internal carotid artery in donkeys using angiographic techniques. This is a prospective descriptive study on 26 cadaveric donkeys.

**Methods:**

Twenty six donkey cadavers of mixed, age, sex and use presented for reasons unrelated to disease of the guttural pouch were subjected to carotid and cerebral angiography using rotational angiography. Rotational angiographic and 3 dimensional multiplanar reconstructive (3D-MPR) findings were verified with an arterial latex casting technique followed by dissection and photography.

**Results:**

The following variations of the carotid arterial tree were identified: [1] the internal carotid and occipital arteries shared a common trunk, [2] the linguofacial trunk originated from the common carotid artery causing the common carotid artery to terminate as four branches, [3] the external carotid artery was reduced in length before giving rise to the linguofacial trunk, mimicking the appearance of the common carotid artery terminating in four branches, [4] the internal carotid artery originated at a more caudal position from the common carotid artery termination.

**Conclusion:**

Veterinarians should be aware that considerable variation exists in the carotid arterial tree of donkeys and that this variation may differ markedly from that described in the horse.

## Background

In horses, the gross and angiographic anatomy of the internal carotid artery has been described [1, 2]. The extracranial pathway of the internal carotid artery begins at the carotid termination where the internal carotid artery originates caudal and ventral to the occipital artery and runs on the dorsal surface of the medial compartment of the guttural pouch. Thereafter the internal carotid artery passes through the foramen lacerum to continue intracranially passing through the ventral petrosal sinus and entering the venous cavernous sinus where the internal carotid artery forms a sigmoid flexure. After the sigmoid flexure, the internal carotid artery gives rise to the caudal intercarotid and caudal communicating arteries before continuing for a short distance rostrally to terminate as the rostral and middle cerebral arteries, [2, 3] forming part of the rostrolateral quadrants of the cerebral arterial circle (Circle of Willis). The caudal communicating artery, which turns caudad to join the basilar artery, results in the formation of the lateral and caudolateral quadrants of the cerebral arterial circle. The objectives of this study were to define the angiographic anatomy of the carotid termination and the internal carotid artery in donkeys; and to determine the variation of the internal carotid artery between individual donkeys using rotational angiographic techniques. To date, carotid and cerebral rotational angiography with 3D imaging (3D-RA) has not been described in donkeys.

## Methods

### Ethics

Ethical approval for this study was granted by the School of Veterinary Medicine Ethics and Welfare Committee, University of Glasgow and consent was obtained for the use of cadaver material.

### Specimen preparation

Twenty six donkey cadavers were obtained. All donkeys that died or were euthanized over a 6 month period regardless of type, age, sex and working purpose were studied. The head and necks were then disarticulated from the body at the level of the 3^rd^ and 4^th^ cervical vertebrae ensuring that the guttural pouches and the carotid arterial tree remained intact on both sides. Signalment data were not recorded during collection of the specimens, but all were adults. The specimens used in this study were not injected with heparin prior to death, thus blood clotting in the vessels at post mortem was to be expected. The muscles surrounding the cervical vertebrae were stripped off to allow identification of the point of disarticulation of the neck from the body. Specimens were collected and kept frozen at -20 °C until they were ready to be transported for the study. Information regarding time interval from death to freezing was unavailable. The specimens were transported (from frozen) using a non-refrigerated vehicle and some degree of thawing had occurred during transportation. Upon arrival the donkey specimens were immediately stored at -20 °C.

The specimens were thawed before any procedures were conducted. The left and right common carotid arteries were identified from the disarticulated region of the neck and catheterised using a 16Fr male Foley catheter advanced into each common carotid artery for approximately 5–10 cm. The balloon on the tip of the catheter was inflated with 1–2 ml of water and an encircling ligature was placed caudal to the balloon to prevent the catheter from becoming dislodged. The arterial system was flushed with water using a high pressure, manual garden pump via the catheter until no resistance was met and water could be seen flowing from the contralateral common carotid artery (via the Foley catheter). This ensured that all the clotted blood in the arteries was removed.

### Rotational angiography and three dimensional multiplanar image reconstruction of the carotid and cerebral vessels

Rotational method of angiography of the carotid and cerebral vessels was carried out using a Ziehm Vario 3D mobile fluoroscopic machine equipped with a C-arm unit. Prior to angiography, the head was placed in left lateral recumbency on a board with two stands to support the weight of the specimen. To extend the guttural pouch, the head was pulled cranially from the neck. Utilising this stand, the specimen was positioned between the X-ray generator and the image intensifier. A laser-positioning beam was directed at the centre of the region cranial to the wings of the atlas, ventral to the ear and caudal to the vertical ramus of mandible. The laser-positioning device aided in the aligning and positioning of the C-arm to the region of interest. A scout radiograph was taken to ensure correct positioning of the C-arm over the region of the guttural pouch and approximately, 15–20 mL of contrast material (Barium sulphate, Baritop ® 100, Sakai Chemical Industry Company Ltd, Japan) was injected to fill the left side of the carotid tree to the level of the internal carotid artery within the cranium. A further 15–20 mL of contrast material was injected to allow filling of the cerebral vessels and the contralateral carotid arterial tree. Following injection of contrast, the Foley catheters on both sides were clamped to prevent leakage of the contrast agent during image acquisition. Angiographic images of the carotid arterial tree were obtained immediately after the ipsilateral injection of the contrast material.

During automatic rotational scanning, the Iso-Cine (for Cine-Loop function) and 3D operating modes (for 3D-multiplanar reconstruction) were used. The C-arm movement was motor-driven and could be controlled using a foot switch, which also controlled exposure. It would take approximately two minutes for the C-arm to complete a 136 degree rotation scan. Each scan generated 112 sequential images at different angles, and these could also be reviewed as a movie using the Cine-Loop function. 3D multiplanar reconstruction (3D-MPR) of the rotational images allowed a 3D representation of the vessels to be created.

### Arterial latex casting, dissection and photography

Angiographic findings were verified using an arterial latex casting technique followed by dissection and photography. For this technique, the arterial system was injected with embalming material (Cambridge formulation, Vickers Laboratory, Pudsey, West Yorkshire, LS28 6QW, UK) via the catheterised common carotid arteries and once the arterial injection was completed, the cadaver was left at ambient temperature for approximately 48 hours to allow the fixative to take effect. Trylon latex (Trylon Ltd, Bury Close, Higham Ferrers Nothants, NN10 8HQ, UK) was used to produce a cast of the carotid arterial tree. After the arterial latex injection, the cadaver was carefully placed in a large heavy-duty container inside a store. The temperature of the storeroom usually depended on the surrounding environmental temperature as the storeroom was not equipped with heating or cooling equipment. Improved air movement in the room was achieved with a fan. Air changes were not monitored. The cadaver was left to allow the latex to cure for 10–14 days. Typically after 10 days, the cadaver was ready for dissection. Photographs of the carotid termination and the extracranial internal carotid artery were taken using a digital camera (Nikon D60 D-SLR) and compared with the angiographic findings.

## Results

### Angiographic variations of the extracranial portion of the internal carotid artery

**Fig. 1:**
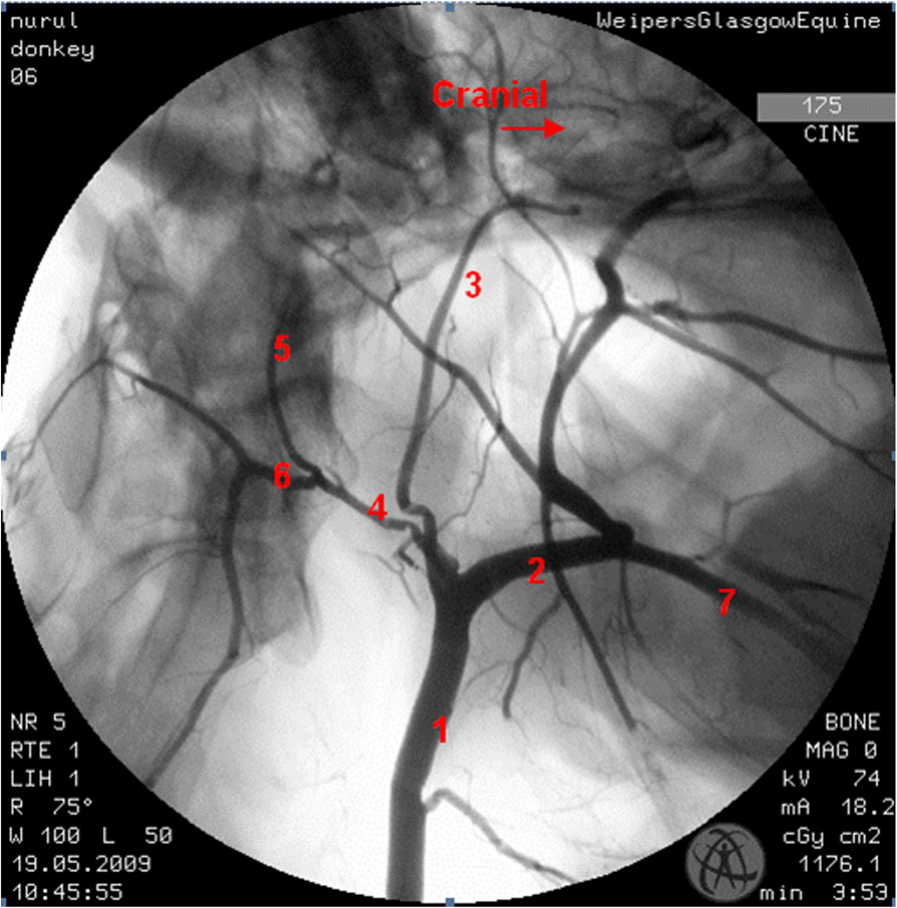
Lateral angiogram (*left*) of the common pattern of the carotid trifurcation and the internal carotid artery of a donkey. 1 common carotid artery; 2 external carotid artery; 3 internal carotid artery; 4 occipital artery; 5 cranial branch of occipital artery; 6 caudal branch of occipital artery; 7 linguofacial trunk

The common/standard anatomical pattern of the extracranial portion of the internal carotid artery and its pathway in donkeys was similar to that observed in the horse (Fig. 1). Rotational angiography of bilateral carotid trifurcation and internal carotid artery was performed on 26 donkeys, producing 52 angiograms. Thirty nine angiograms demonstrated a pattern common to that found in horses [1] and 13 had anatomical variations.

**Fig. 2:**
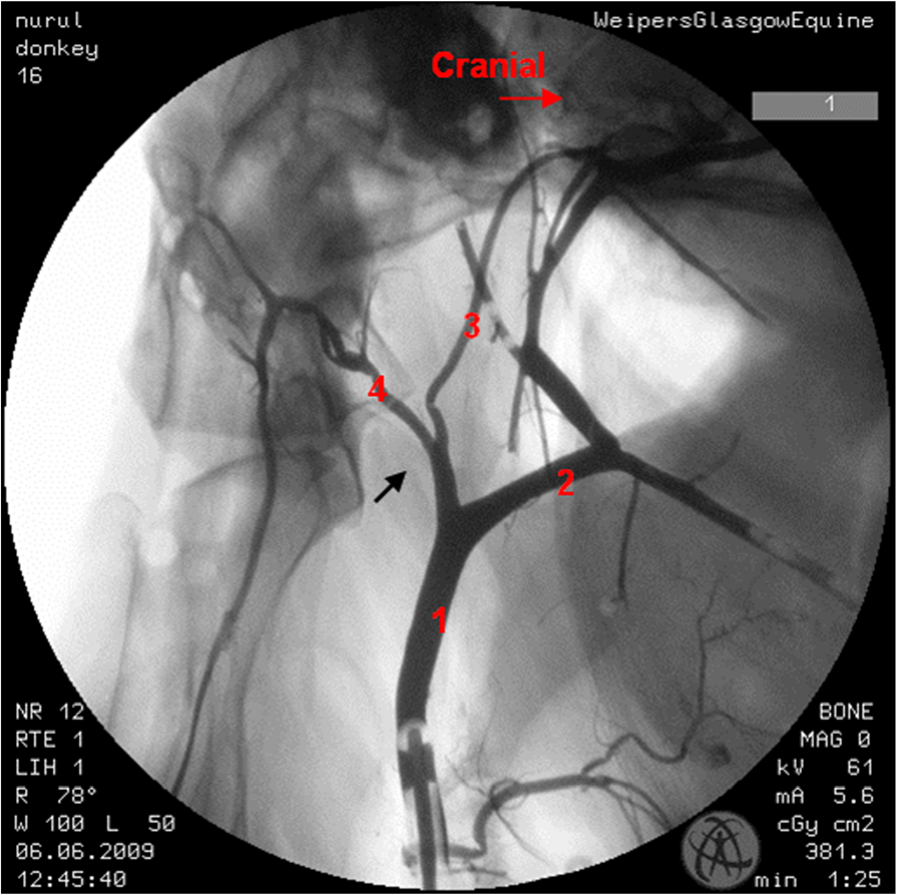
Lateral angiogram of the *left* carotid arterial tree of a donkey shows variation from the common pattern of this structure where the occipital and the internal carotid arteries share a common trunk (*black arrow*). 1 common carotid artery; 2 external carotid artery; 3 internal carotid artery; 4 occipital artery

**Fig. 3:**
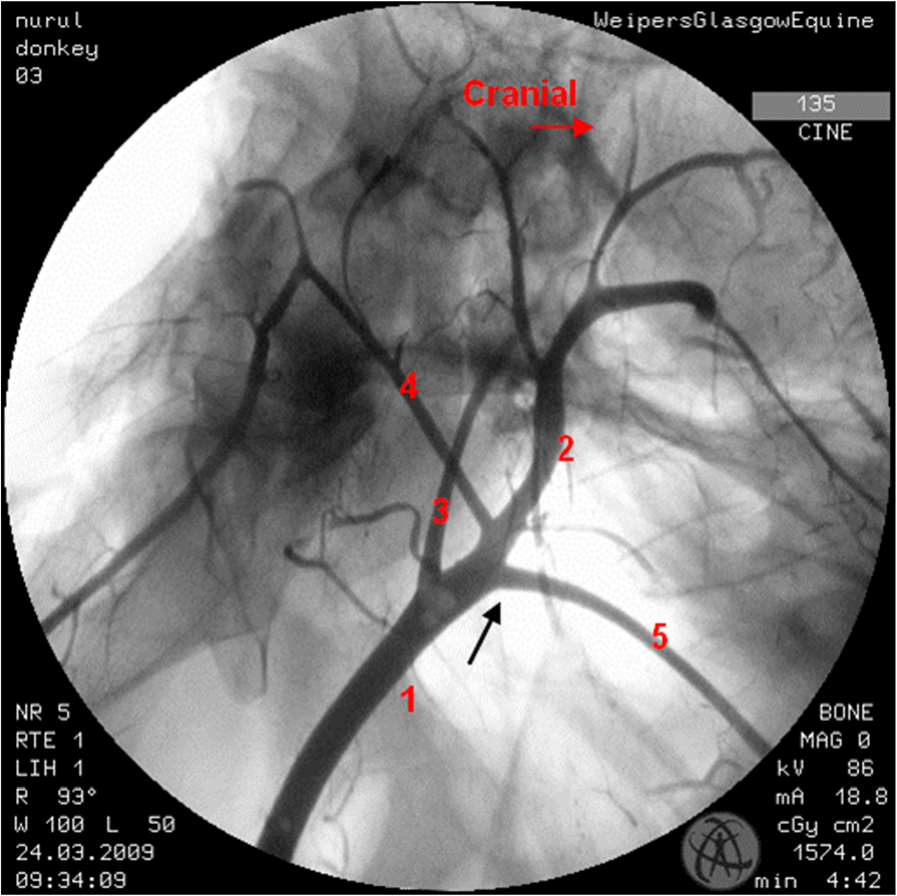
Lateral angiogram of the *left* carotid arterial tree of a donkey shows variation from the common pattern of this structure. The origin of the linguofacial trunk (*black arrow*) is directly from the common carotid artery. 1 common carotid artery; 2 external carotid artery; 3 internal carotid artery; 4 occipital artery; 5 linguofacial trunk

**Fig. 4:**
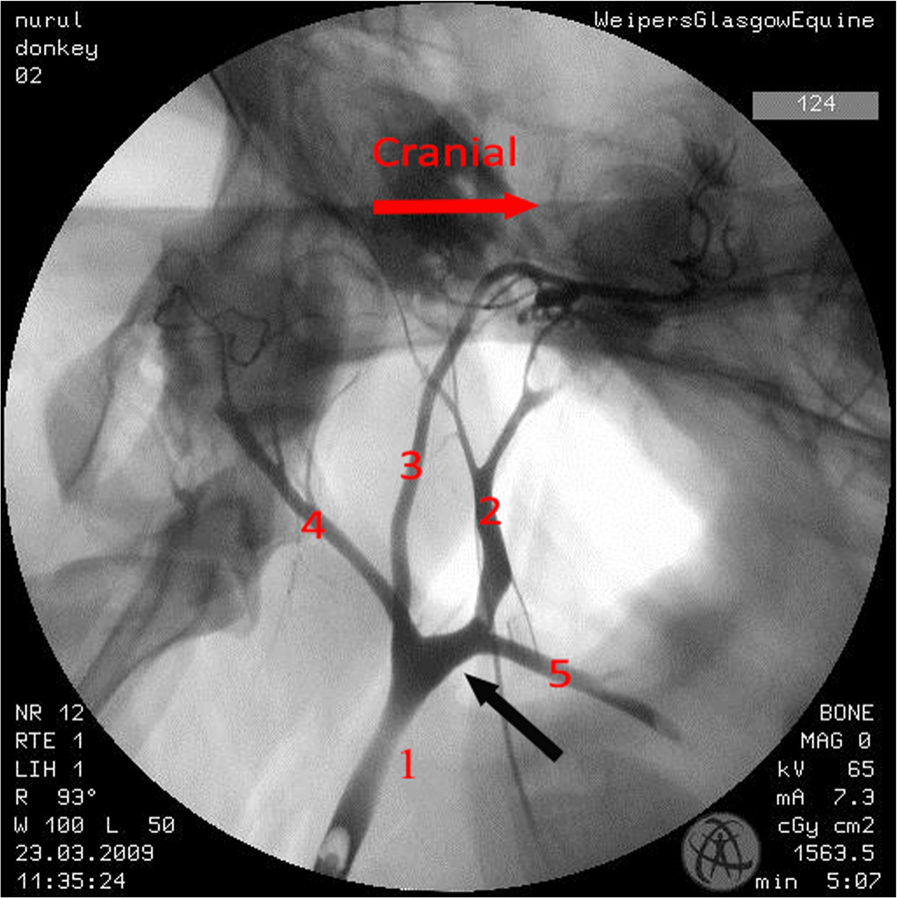
Lateral angiogram of the *left* carotid arterial tree of a donkey shows variation from the common pattern of this structure. The linguofacial trunk shares the same origin with the external carotid artery (*black arrow*). 1 common carotid artery; 2 external carotid artery; 3 internal carotid artery; 4 occipital artery; 5 linguofacial trunk

Where there were differences in the angiographic appearance in comparison to horses, these variations affected the termination of the common carotid artery. Sharing of a common trunk with the occipital and the internal carotid arteries was observed unilaterally in one donkey (Fig. 2). Five angiograms demonstrated the linguofacial trunk originating from the common carotid artery (Fig. 3). In another five angiograms, it appeared that the linguofacial trunk shared a common origin with the external carotid artery (Fig. 4).

**Fig. 5:**
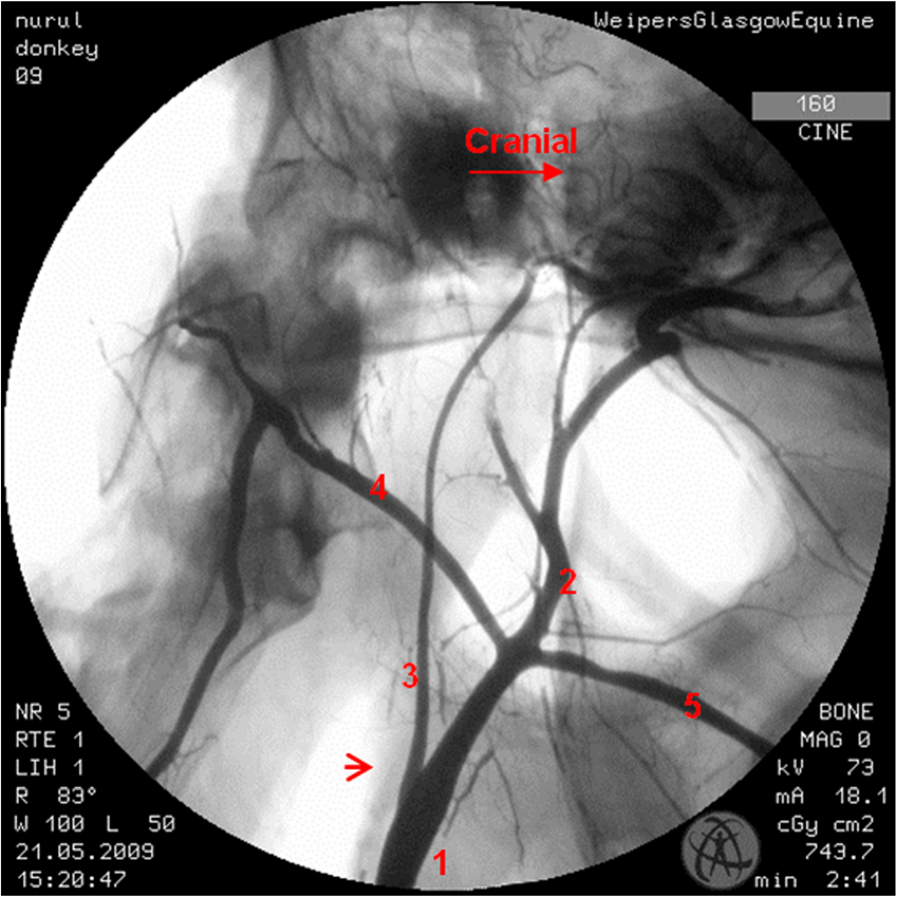
Lateral angiogram of the *left* carotid arterial tree of a donkey shows variation from the common pattern of this structure. The *left* internal carotid artery (*red open arrow*) originates very caudal to the common carotid artery termination. 1 common carotid artery; 2 external carotid artery; 3 internal carotid artery; 4 occipital artery; 5 linguofacial trunk

Another interesting finding in two angiograms was that the internal carotid artery originated much more caudal to the occipital artery (Fig. 5), compared to the common anatomical pattern. The distance of the internal carotid artery origin from the common carotid artery was neither measured nor recorded during angiography, but was measured during dissection. Bilateral variations were noted in one donkey, and unilateral variations in 11 donkeys. Of 13 angiograms with variations at the termination of the carotid arterial tree, it was noted that nine variations were on the left side and four were on the right.

### Angiography of the intracranial portion of the internal carotid artery and the cerebral arterial circle

**Fig. 6:**
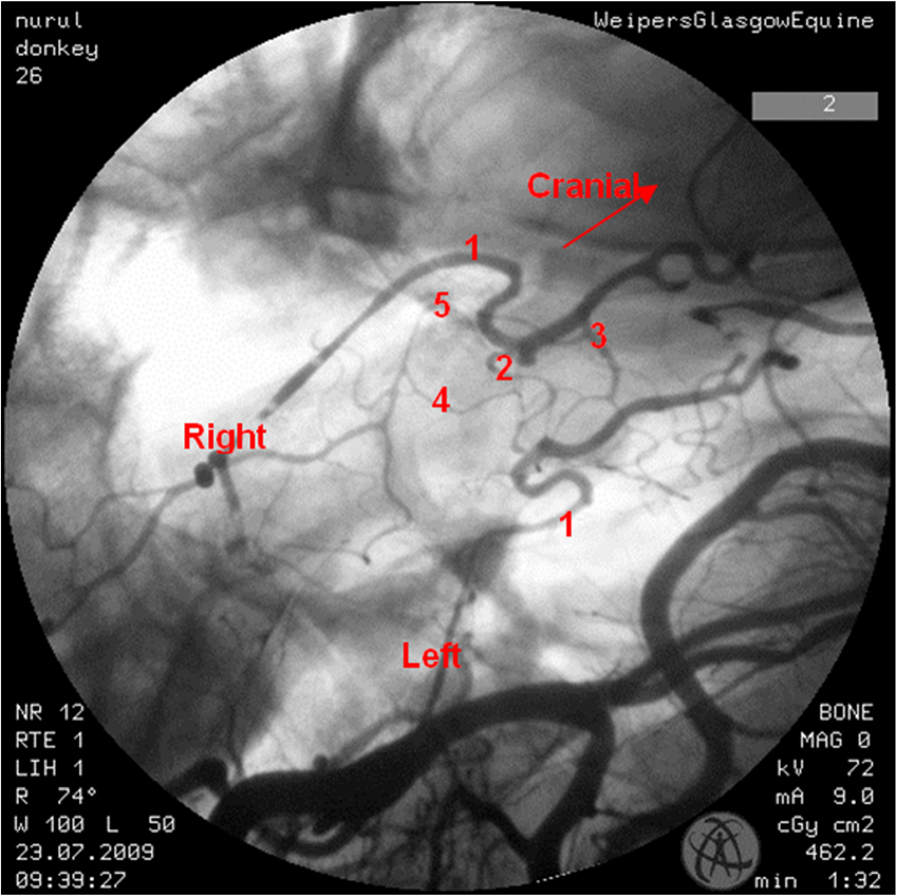
Dorsoventral angiogram of the cerebral arterial circle of a donkey. The basilar artery was not straight and leaning more to the *right side*. Note the presence of the right caroticobasilar artery (arising from the second *curve* of the internal carotid artery). 1 internal carotid artery; 2 intercarotid artery; 3 caudal communicating artery; 4 basilar artery; 5 caroticobasilar artery

**Fig. 7:**
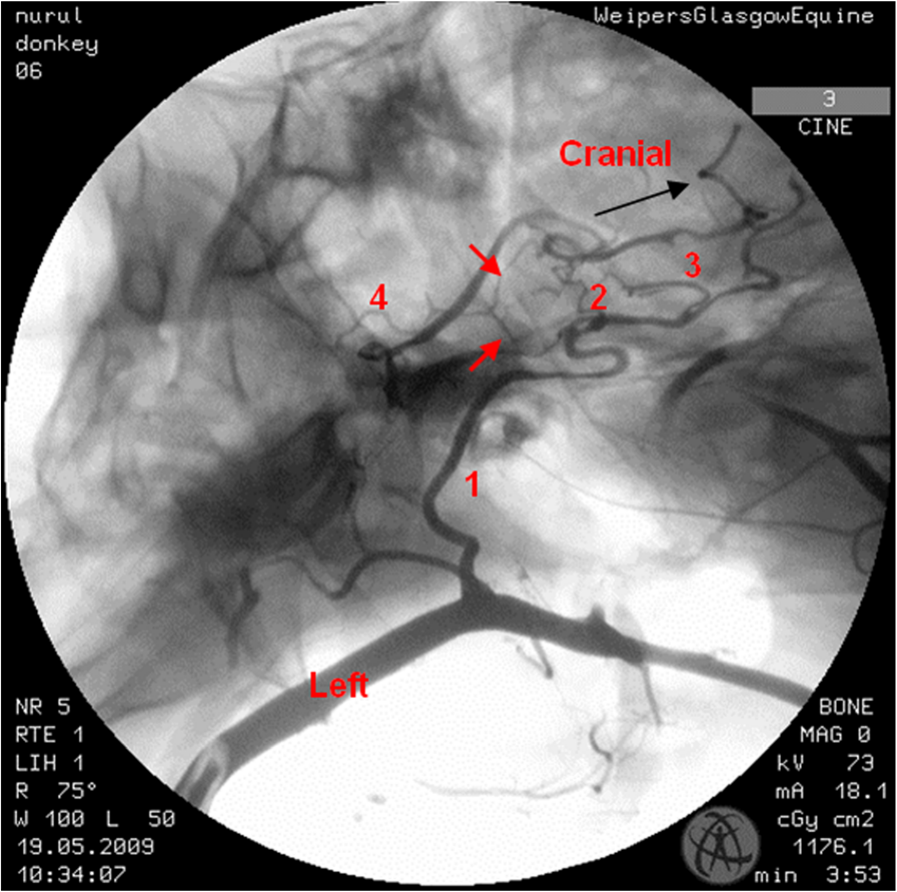
Dorsoventral angiogram of the common pattern of the internal carotid arteries and formation of the cerebral arterial *circle* of a donkey. Note the bilateral presence of caroticobasilar arteries (*red arrows*). 1 internal carotid artery; 2 intercarotid artery; 3 caudal communicating artery; 4 basilar artery

**Fig. 8:**
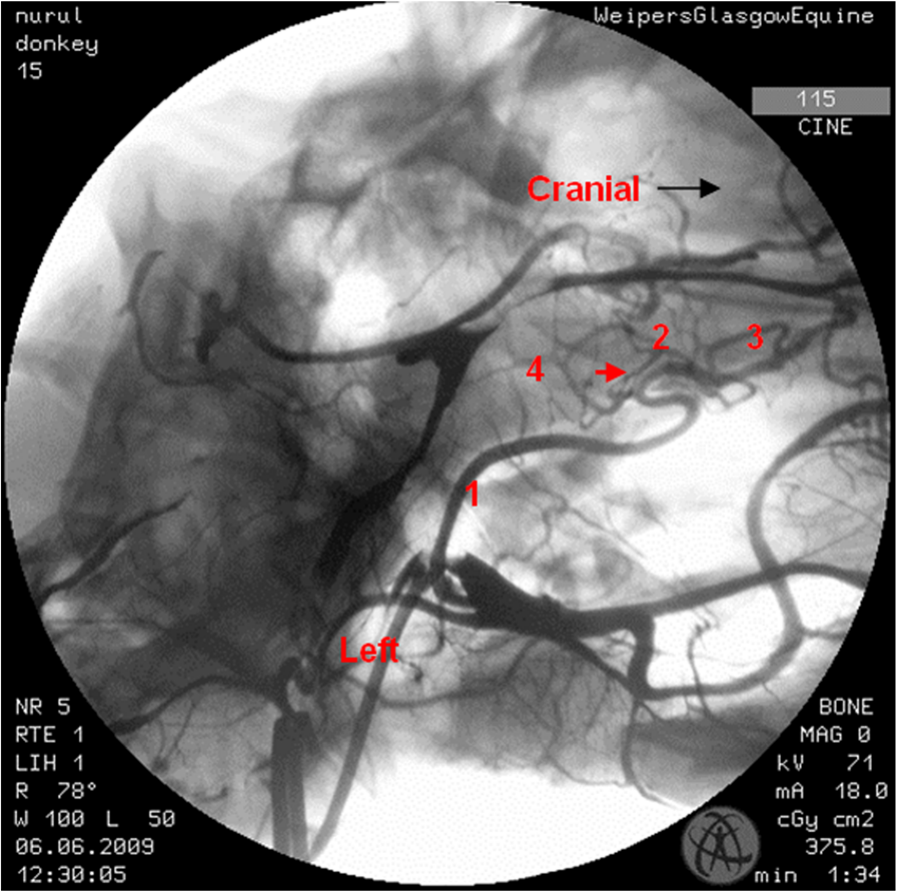
Dorsoventral angiogram of the cerebral arterial *circle* of a donkey. A peculiar connection is seen (*red arrow*) from the second *curve* of sigmoid flexure of the internal carotid artery to the caudal intercarotid artery. 1 internal carotid artery; 2 intercarotid artery; 3 caudal communicating artery; 4 basilar artery

**Fig. 9:**
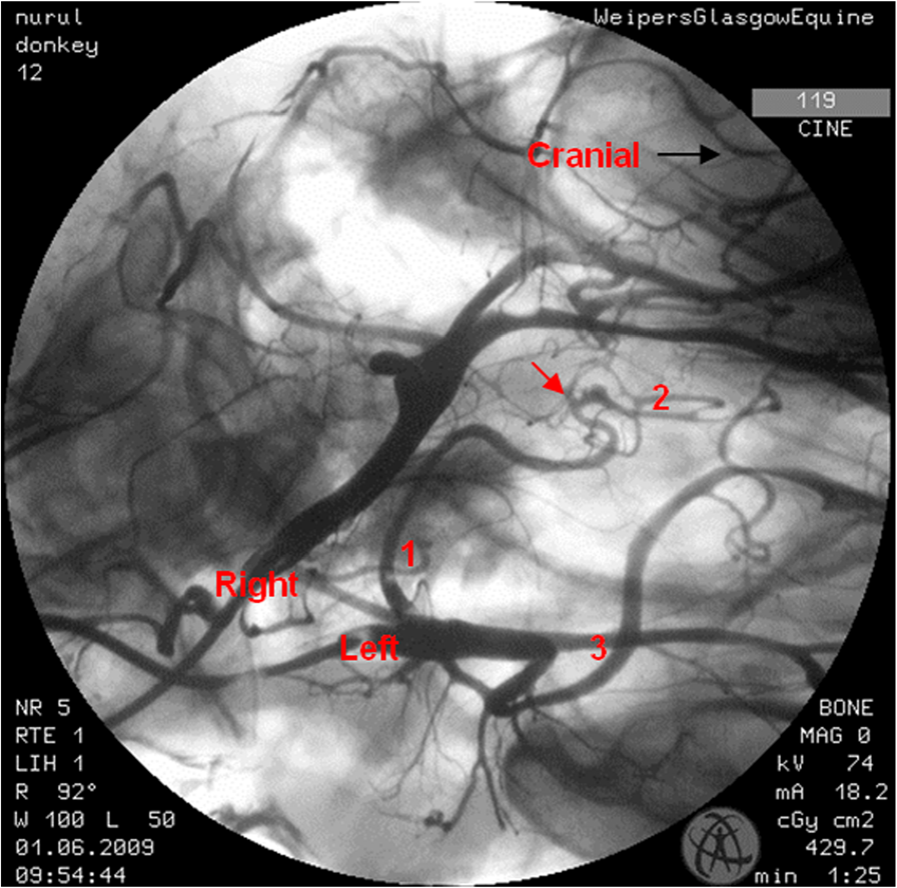
Dorsoventral angiogram of the cerebral arterial *circle* of a donkey. A connection is seen from the second *curve* of the *right* internal carotid artery to the caudal communicating artery (*red arrow*). 1 internal carotid artery; 2 caudal communicating artery; 3 external carotid artery

In the region of the intracranial portion of the internal carotid artery, the presence of the caroticobasilar arteries could be appreciated in 20 donkeys, either unilaterally (10) or bilaterally (10). Figure 6 shows an example of the unilateral presence of this artery and Fig. 7 a bilateral example. However, several eccentric connections were seen in addition to this artery in a number of donkeys (3/26). In two donkeys, a small vessel originating from the second curve of the sigmoid flexure of the left internal carotid artery was noted to be connected to the caudal intercarotid artery (Fig. 8). In another donkey, an eccentric connection was seen on the left side where a vessel originating from the second curve of the sigmoid flexure was observed to join the caudal communicating artery before that artery joined the basilar artery to form the caudolateral quadrants of the cerebral arterial circle (Fig. 9). Unfortunately, it was difficult to determine whether this was a true vessel or superimposition with other vessel. In one donkey, it was thought that the caudal intercarotid artery was absent; however its presence was confirmed with repeated views using the Cine-Loop function.

### Dissection of the latex casted specimens

Dissections were carried out on 26 donkey specimens that had been subjected to latex casting of the carotid arterial tree confirming the angiographic findings in all the specimens at the level of extracranial part of the carotid arterial tree. In the 26 donkeys where bilateral carotid arterial tree latex casting was attempted, only two satisfactory casts of the carotid arterial tree were obtained out of 52 casts. Where the casting technique was unsuccessful, it appeared that the embalming fluid and latex failed to fill the arterial lumen satisfactorily, resulting in shrinking of the samples (25 casts). However, partially filled casts were nevertheless dissected as in some cases they retained enough shape to be of value (3 casts). Twenty two latex casts from non-embalmed specimens were not harvested. An immediate anatomical observation on these specimens was made and recorded during dissections.

## Discussion

In the donkey, the angiographic appearance of the carotid trifurcation and the internal carotid artery has been reported to have a similar pattern to that of the horse [4]. However, one major difference was that the common carotid artery may terminate as four branches instead of three, where the linguofacial trunk (external maxillary artery) arises directly from the common carotid artery and not as a branch of the external carotid artery [4]. Another difference noted was that the occipital artery of donkeys was larger than in horses [4].

### Angiography of the extracranial portions of the internal carotid artery

Previously published angiographic studies of the carotid arterial tree of the donkey, described the origin of the linguofacial trunk as the common carotid artery, and not as a branch of the external carotid artery, as is the case in the horse [4]. However, this observation was based on a single donkey, thus this finding cannot be regarded as conclusive. Based on the study presented here, it appears that the carotid trifurcation of the donkey is similar to that of the horse (based on 39/52 angiograms). However, a number of the donkey cadavers (13/52) showed the following variations:

1. The internal carotid artery and occipital artery shared a common trunk (1/13 angiograms)
2. The linguofacial trunk was present as a branch of the common carotid artery, rather than as a branch of the external carotid artery (5/13 angiograms)
3. The linguofacial trunk shared a common trunk with the origin of the external carotid artery (5/13 angiograms)
4. The internal carotid artery originated more caudally, than the usual pattern, from the common carotid artery termination (2/13 angiograms)

In this study, the origin of the internal carotid artery was observed to be commenced more caudal than the commonly understood anatomical pattern of this structure in two of the cadavers. Usually, the internal carotid artery originated very close to the occipital artery. In donkeys that have a linguofacial trunk originating from the common carotid artery and an internal carotid artery originating very caudal to the termination of the common carotid, there may be confusion in identifying the internal carotid artery, given the assumption that the common carotid artery terminates in three branches. One may mistakenly think that the internal carotid artery was absent, or worse, identify the occipital artery as the internal carotid artery.

The anatomical arrangement of the linguofacial trunk may create confusion in terms of where exactly this vessel originates. We speculate that if the linguofacial trunk is given off close to the origin of the external carotid artery it may appear angiographically, that the two vessels share a common origin.

In this study, a number of anatomical variations present in the donkeys have been previously described in horses as well. However, interestingly, aberrant branches of the internal carotid artery, as noted in horses [1, 4–6], were not observed in any of the donkeys in this study.

### Angiography of the intracranial portions of the internal carotid artery

There is a paucity of published literature relating to the internal carotid artery and the cerebral arterial circle of donkeys. In one report [7] it was argued that the internal carotid artery bifurcates intracranially into caudal communicating and rostral cerebral arteries, with the middle cerebral artery branching from the rostral cerebral artery and not from the internal carotid artery. Contrary to what has been described in the horse, the internal carotid artery was considered to give off the caudal communicating artery and continue for a short distance rostrally to terminate as the rostral and middle cerebral arteries [2]. In other words, the rostral cerebral artery starts after the origin of the middle cerebral artery.

A rostral intercarotid artery was observed in the cerebral arterial circle in one donkey [7]. This was described as a thin vessel originating from the extension of the internal carotid artery (perhaps more correctly described as the rostral cerebral artery) at the level where the internal carotid artery gives off the caudal communicating artery. The rostral intercarotid artery formed a connection to the contralateral vessel.

The description given for the caudal communicating artery in the horse, is that the artery turns caudal after coming off the intracranial portion of internal carotid artery, and then joins the basilar artery to form the lateral and caudolateral quadrants of the cerebral arterial circle [2]. In this study, generally the arrangement of the caudal communicating giving rise to the basilar artery followed this pattern. However in some donkeys, it was observed that there were some indirect fine branches at the terminal end of the caudal communicating arteries, and generally the continuation to the basilar artery was not easy to determine. The junction of the distal segment of the caudal communicating artery with the basilar artery is created by a rete or plexus of the terminal branches of the basilar artery as recognised by Sisson (1910). According to Nanda [2], a rostral cerebellar artery originates from the terminal portion of the basilar artery, of which there can be either two or three on either side. Where a plexus or rete can be seen before the basilar artery joins the caudal communicating artery, the rostral cerebellar artery may leave this plexus in a variable and asymmetrical manner [2].

The odd arrangement of the caudolateral quadrants of the cerebral arterial circle has also been reported [8]. It was suggested that the caudal communicating artery should not be named as such because of the presence of various fine branches interconnected with the basilar artery, which were arranged in an odd manner similar to the primitive patterns observed in other lower mammals and submammals (fishes, amphibians and reptiles). Perhaps in view of the results described here, the variations in arrangement of these fine vessels might be regarded as variant anastomoses of the cerebral arterial circle. Based on the observations herein, the rete was seen as various anastomotic fine vessels interconnecting the basilar artery, not only to the caudal communicating but also to the caudal intercarotid arteries, as also seen in the angiographic findings of the donkeys studied here. Due to the complex three dimensional structures of the cerebral arterial circle and its connections, the best way to verify the continuation of these eccentric vessels is by viewing its three dimensional display using the 3D-MPR function. However, due to the fact that the 3D-MPR representations faced some limitations of its own, further investigation on these eccentric vessels could not be carried out accordingly.

## Conclusion

The findings described here, provide additional information regarding the pathway of the extracranial and intracranial internal carotid artery pathway. The intracranial internal carotid and the cerebral arterial circle could be observed angiographically and these findings complement the anatomical study of the blood supply to the brain of donkeys.

## Abbreviations

3D- MPR: 3 dimensional multiplanar reconstructive
3D- RA: 3 dimensional rotational angiography

## References

[1] Khairuddin NH, Sullivan M, Pollock PJ (2015). Angiographic variation of the internal carotid artery, and its branches, in horses. Vet Surg.

[2] Nanda BS, Sisson S (1975). Heart and arteries. The Anatomy of the Domestic Animals.

[3] Nanda BS, Getty R (1975). Presence of the arteria caroticobasilaris in the horse. Anat Anz.

[4] Colles CM, Cook WR (1983). Carotid and cerebral angiography in the horse. Vet Rec.

[5] Freeman DE, Staller GS, Maxson AD, Sweeney CR (1993). Unusual internal carotid artery branching that prevented arterial occlusion with a balloon-tipped catheter in a horse. Vet Surg.

[6] Lepage OM (2005). Transarterial coil embolisation in 31 horses (1999-2002) with guttural pouch mycosis. Equine Vet J.

[7] Ozgel O, Dursun N (2007). Arteries that supply the brain and the formation of circulus arteriosus cerebri in donkeys. Med Weter.

[8] Gillilan LA (1974). Blood supply to brains of ungulates with and without a Rete Mirabile Caroticum. J Comp Neurol.

